# Determination of Ploidy Level and Isolation of Genes Encoding Acetyl-CoA Carboxylase in Japanese Foxtail (*Alopecurus japonicus*)

**DOI:** 10.1371/journal.pone.0114712

**Published:** 2014-12-11

**Authors:** Hongle Xu, Wenpan Zhang, Teng Zhang, Jun Li, Xian Wu, Liyao Dong

**Affiliations:** 1 College of Plant Protection, Nanjing Agricultural University, Nanjing, China; 2 Key Laboratory of Integrated Pest Management on Crops in East China (Nanjing Agricultural University), Ministry of Agriculture, Beijing, China; 3 National Key Laboratory of Crop Genetic & Germplasm Enhancement, Cotton Research Institute, Nanjing Agricultural University, Nanjing, China; University of Umeå, Sweden

## Abstract

Ploidy level is important in biodiversity studies and in developing strategies for isolating important plant genes. Many herbicide-resistant weed species are polyploids, but our understanding of these polyploid weeds is limited. Japanese foxtail, a noxious agricultural grass weed, has evolved herbicide resistance. However, most studies on this weed have ignored the fact that there are multiple copies of target genes. This may complicate the study of resistance mechanisms. Japanese foxtail was found to be a tetraploid by flow cytometer and chromosome counting, two commonly used methods in the determination of ploidy levels. We found that there are two copies of the gene encoding plastidic acetyl-CoA carboxylase (ACCase) in Japanese foxtail and all the homologous genes are expressed. Additionally, no difference in ploidy levels or ACCase gene copy numbers was observed between an ACCase-inhibiting herbicide-resistant and a herbicide-sensitive population in this study.

## Introduction

Ploidy level, defined as the number of sets of chromosomes in the nucleus, is an important genomic characteristics in biodiversity studies and developing strategies for isolating important plant genes [1], [2], [3]. Although knowledge on ploidy levels continues to improve in angiosperms, little is known regarding those of weeds. Nevertheless, it has been shown that polyploidy is more frequent in weeds than in other species, especially in natural area invaders and agricultural weeds [4], [5]. The advantages of being polyploidy may predispose such weeds to be biologically invasive species [5], [6]. The intensive and global use of herbicides to control agricultural weeds has resulted in the evolution of resistance, and herbicide-resistant weeds pose a great threat to global agriculture. To date, resistance is documented in 431 resistant biotypes in 235 species [7]. Furthermore, compared with the knowledge of diploid weeds, our understanding of the evolution of herbicide resistance in polyploid weeds is limited [8].

Acetyl-CoA carboxylase (ACCase; EC 6.4.1.2) is a key enzyme in most organisms, catalyzing the carboxylation of acetyl-CoA to form malonyl-CoA [9], [10]. Plants have two ACCase isoforms [11], [12]. Cytosolic ACCase, encoded by a single nuclear gene, is a multidomain and homodimeric enzyme in all plants. Plastidic ACCase is a multisubunit and heteromeric enzyme consisting of three nuclear-encoded subunits and one plastidic-encoded subunit in most plants [11], [12], [13]. The exception is the plastidic ACCase in the Poaceae family, which is a multidomain and homodimeric enzyme, encoded by a nuclear gene [11], [12], [13], [14]. The difference among plastidic ACCase between broadleaf and grass weeds is the basis for the selectivity of ACCase-inhibiting herbicides [9]. The cytosolic ACCase and the plastidic heteromeric ACCase are relatively insensitive, but the plastidic homomeric ACCase is sensitive to ACCase-inhibiting herbicides [9], . The continuous and extensive use of these herbicides has resulted in resistance evolution in at least 46 weeds [7]. Two mechanisms known to confer ACCase-inhibiting herbicide resistance to plants are target site resistance (TSR) and non-target site resistance [14], [15], [16], [17]. To date, substitutions at seven codon positions: (1781, 1999, 2027, 2041, 2078, 2088 and 2096) in the carboxyl transferase domain of plastidic ACCase genes have been identified to endow resistance to ACCase-inhibiting herbicides [18], [19], [20], [21], [22]. However, the ploidy level of many weeds is unknown. This may influence the isolation of target genes of interest since the ploidy level of the weed will determine the number of target genes. Thus, determining the ploidy of weeds is meaningful in studying resistance.

Japanese foxtail (*Alopecurus japonicus* Steud.), an annual weed of the Poaceae family, is one of the most noxious weeds infesting cereal and oilseed rape fields in China and Eastern Asia [23]. Resistance to ACCase and acetolactate synthase (ALS)-inhibiting herbicides has been documented in Japanese foxtail [24], [25], [26], [27], [28], [29]. Although Japanese foxtail is one of the most studied herbicide-resistant grass weeds in China, most of the previous studies ignored the multiple copies of target genes. The traditional method for determining ploidy level is chromosome counting, which has proven reliable in many different species [30], [31]. Flow cytometry, a convenient, fast and reliable method, has also been used recently to determine the DNA content and ploidy levels in many plant species [32]. The objectives of this study are to determine the ploidy level of Japanese foxtail and to assess any difference in this respect between a sensitive and resistant population.

## Materials and Methods

### Plant materials

Seeds from a resistant (R) Japanese foxtail population JLGY-4 were collected in 2011 from a wheat field in Ganyu, Lianyungang, Jiangsu province in China (34°78′N, 119°12′E). The R population evolved resistance to the ACCase-inhibiting herbicides fenoxaprop-P-ethyl and pinoxaden [29]. A sensitive (S) population (JLGY-1) was collected near the R population on a river bank that had never been treated with herbicide (34°78′N, 119°06′E). This population has been documented to be sensitive to ACCase-inhibiting herbicides [29]. The distance between the two sites is approximately 5 km. Seeds from both R and S populations were sown in 12-L pots filled with a 2:1 (wt/wt) mixture of sand and soil, and seedlings were grown in the greenhouse. The growth conditions were 25/20°C day/night temperatures (± 3°C) and 12/12 h cycles of light/dark with a light intensity of 450 µmol photons m^–2^ s^–1^. All plants were actively growing and healthy at the time of the sampling. Both R and S plants were used in the following study.

“No specific permissions were required for the location where the Japanese foxtail seeds were collected. This study did not involve any endangered or protected species”.

### Flow cytometry

Eight samples from each of R and S populations were analyzed by flow cytometry. Flow cytometry was performed essentially as described by Dolezěl et al. [32]. Briefly, approximately 100 mg of fresh Japanese foxtail leaf tissue was harvested and transferred to a glass Petri-dish (on ice). Approximately 80 mg of fresh perennial ryegrass (*Lolium perenne* L., 2n  =  4X  =  28) leaf tissue served as an external reference standard. Tissues were finely chopped with a razor blade in ice-cold LB01 lysis buffer (15 mM Tris, 2 mM Na_2_EDTA, 0.5 mM spermine tetrahydrochloride, 80 mM KCl, 20 mM NaCl, and 0.1% (v/v) Triton X-100) [33]. After chopping, the suspension was filtered through a 50-µm nylon mesh, and RNase A and propidium iodide (both to 50 mg/ml final concentrations) were added. A suspension of isolated nuclei was then incubated on ice in darkness for 30 min prior to analysis.

Flow cytometry was performed with an Accuri C6 flow cytometer (BD Accuri, USA). For each sample, 5000 to 10,000 nuclei were collected and analyzed. The results were displayed as one-parameter DNA histograms (G_0_/G_1_ peak). Flow cytometer records the fluorescence intensity of cells in G_0_/G_1_ and G2 periods. Cells in the G_0_/G_1_ period precede DNA synthesis. Cells in the G_2_ period have finished DNA replication but have not divided. Thus, the ploidy level of the sample can be determined by the equation [32]: Sample ploidy  =  reference ploidy × (mean position of sample G_0_/G_1_peak)/(mean position of standard G_0_/G_1_peak)

### Chromosome counts preparation and cytological measurement

Japanese foxtail root tips were harvested from germinated seeds, pretreated with 2 mM 8-hydroxyquinoline for 4–6 h at 20°C to accumulate metaphase cells, and fixed in methanol–acetic acid (3:1) fixative. Root tips were macerated in 1 M HCl at 20°C for 1h and squashed in 45% acetic acid. All slides were stored at 70°C. After removing the coverslips, slides were dehydrated using 100% ethanol prior to the chromosome counting. Slides were stained in 4,6-diamidino-2-phenylindole (Roche Diagnostics, Switzerland) for 5 min at room temperature, and finally, anti-fade (Vector, USA) was applied under the coverslip. Slides were examined under an Olympus BX51 fluorescence microscope. Chromosome images were captured using an Evolution VF CCD camera (Media Cybernetics, USA) and merged using Image-Pro Express software.

### DNA isolation and Southern blot analysis

Southern blotting was carried out to confirm the existence of multiple copies of genes encoding plastidic ACCase in Japanese foxtail. The genomic DNA of Japanese foxtail was isolated from the young leaf tissues of R and S individuals according to Xu et al. [24]. Southern blot analysis was performed according to the manufacturer's instructions using the DIG High Prime DNA Labeling and Detection Starter Kit I (Roche Diagnostics). In brief, 30 µg of genomic DNA was digested with *Hae*III, *Eco*RI and double digested with both enzymes, and then electrophoresed through a 1.0% agarose gel. DNA was transferred onto Pure Nitrocellulose Blotting Membranes (Solarbio Science&Technology Co., China) by capillary transfer and crosslinked under UV light. The hybridization probe labeled with digoxigenin was a 553 bp PCR fragment amplified by the primer pair ACCp1F/ACCp1R (Table 1). After hybridization, the membrane was washed and analyzed following the manufacturer's instructions.

**Table 1:** List of primers.

Primer	Sequence (5′-3′)	Usage	Product size (bp)	Annealing temperature (°C)	References
ACCp1F	GCAAACTCIGGTGCTCGGATTGGCA	Southern probe and sequencing	553	60	[29]
ACCp1R	GAACATAICTGAGCCACCTCAATATATT				
ACCp2F	TGCATACAGCGTATTGACCAG	Sequencing	873	60	[29]
ACCp2R	CTCTGACCTGAACTTGATCTC				

### Total RNA extraction, cDNA synthesis and PCR amplification

Total RNA was extracted from 100 mg young shoot tissue of individuals using RNAiso Plus (TaKaRa Biotech, China) according to the manufacturer's instructions. Genomic DNA contamination was removed using the Recombinant DNase I (TaKaRa). cDNA was synthesized following the manufacturer's instructions using a PrimeScript 1st Strand cDNA Synthesis Kit (TaKaRa). The primer pairs ACCp1F/ACCp1R and ACCp2F/ACCp2R were used to amply a 1230 bp region containing the resistance-endowing ACCase gene mutation sites (Table 1) [29]. The PCR procedure was conducted as described in Xu et al. [24]. Purification of the PCR products, cloning and sequencing protocols were as described previously [29]. At least eight clones for each biological replicate were sequenced. BioEdit Sequence Alignment Editor software was used to align and compare the sequence data.

## Results

### Flow cytometry

**Figure 1:**
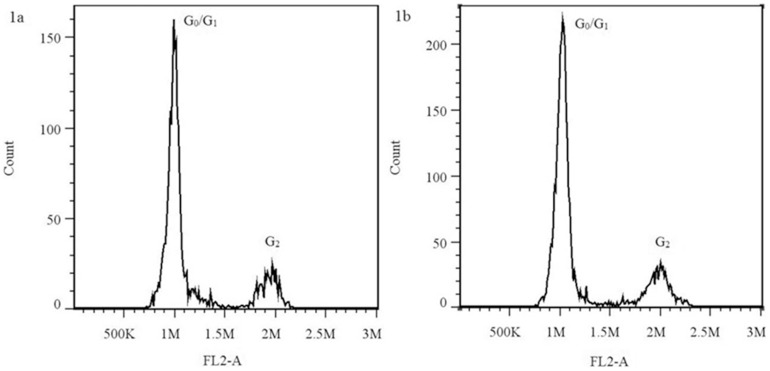
Flow cytometric analysis of homogenates prepared from perennial ryegrass (a) and Japanese foxtail (b).

The mean fluorescence value of the G_0_/G_1_ peak positions for the reference standard was 9.84×10^5^. The mean fluorescence values of the G_0_/G_1_ peak positions for the R and S samples were 1.01×10^6^ and 1.00×10^6^, respectively. The coefficient of variation of the cytometry values, as a parameter of the reliability of the measurement, varied between 2.3% and 4.7%. These values were considered acceptable [32]. The flow cytometry results revealed that all these samples were tetraploid. Thus, Japanese foxtail is a tetraploid grass weed species (Fig. 1).

### Chromosome count

**Figure 2:**
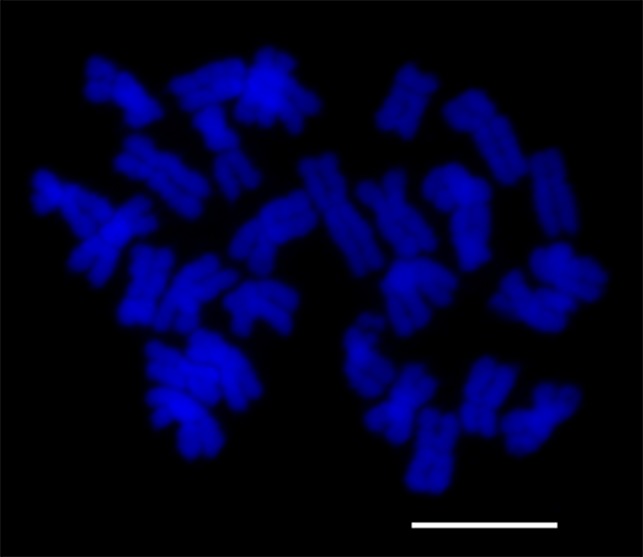
Mitotic metaphase chromosomes of Japanese foxtail. Scale bar is 10 µm.

Mitotic metaphase chromosomes are advantageous for counting chromosomes. As previously reported, chromosome numbers were used to confirm the ploidy levels of different species. To determine the ploidy level of Japanese foxtail, chromosome counting was used to corroborate the flow cytometry results. More than 20 excellent mitotic cells with dispersed metaphase chromosomes were obtained and counted. The result showed 28 chromosomes in each cell (Fig. 2). Interestingly, because of the basic chromosome number in *Alopecurus*, which is X  =  7, the result is in accordance with 2n  =  4X  =  28 [34]. Thus, Japanese foxtail was confirmed to be tetraploid.

### Southern blot analysis

**Figure 3:**
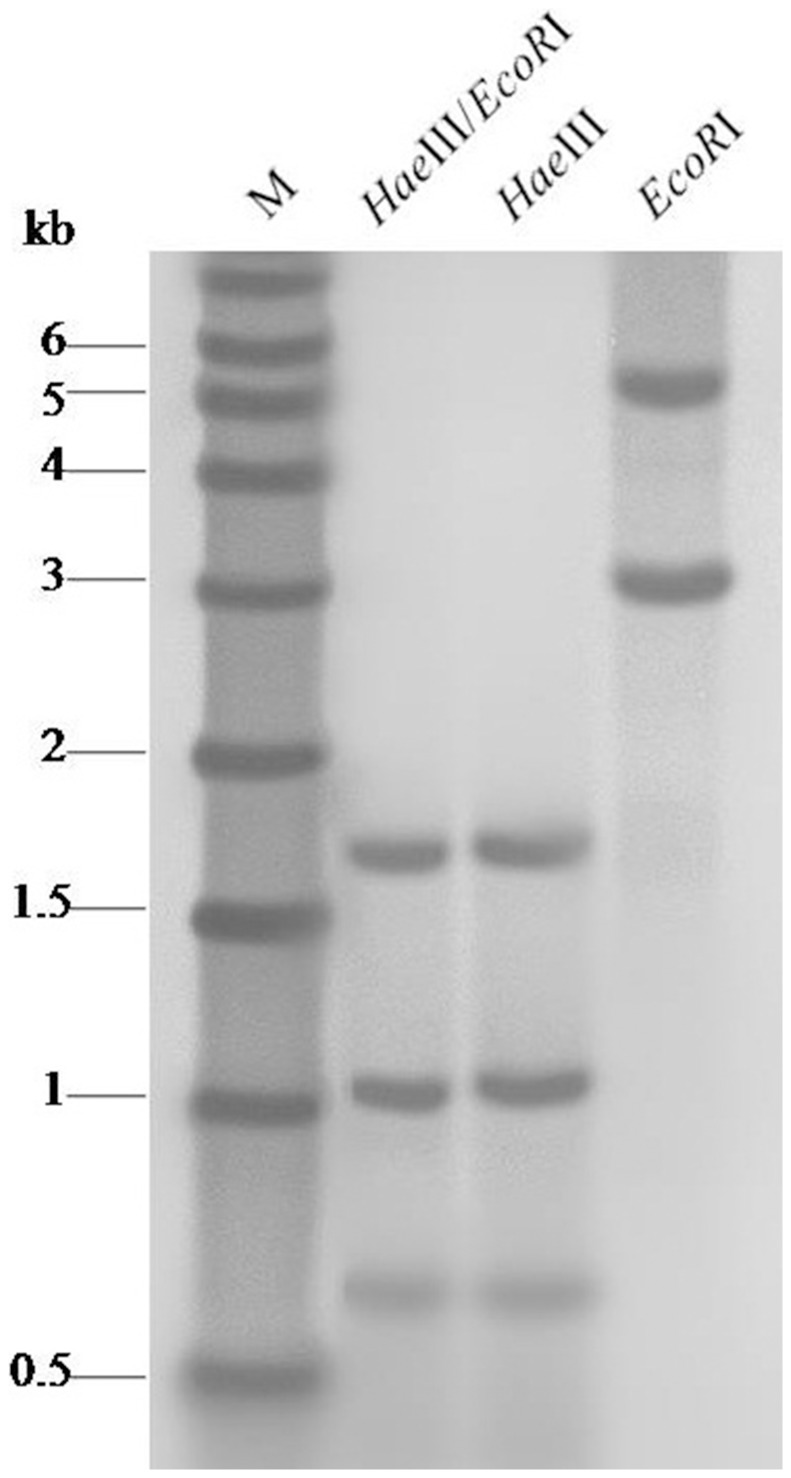
Genomic southern blot hybridization analysis of Japanese foxtail digested with *Eco*RI, *Hae*III and both enzymes. M, DIG labeled DNA molecular weight.

A genomic hybridization analysis was performed to estimate the copy number of the gene encoding plastidic ACCase in Japanese foxtail. The hybridization pattern after digestion with *Eco*RI, *Hae*III or both enzymes displayed two, three and three fragments, respectively (Fig. 3). The same hybridization pattern was observed in the R and S populations of Japanese foxtail (data not shown). Since there is no restriction site for either enzyme within the probe, these data suggest that at least two copies of the ACCase gene should be present in the Japanese foxtail genome. The presence of multiple copies of the ACCase gene would be in accordance with the tetraploid nature of Japanese foxtail. This result confirmed the previous finding by our group [29].

### ACCase gene isolation and sequencing

Since two copies of the ACCase genes have been isolated previously, this study was conducted to confirm that both of the homologous genes isolated are expressed. Reverse transciptase-PCR analysis revealed that two homologous genes can be transcribed. The isolated cDNA lengths of *Acc1;1* and *Acc1;2* were all 1230 bp (GenBank accession numbers KJ781292 and KJ781293 for the S population, and KJ781294 and KJ781295 for the R population). No intron was found for either gene when comparing the genomic sequences obtained previously. The deduced amino acid sequences of *Acc1;1* and *Acc1;2* showed 99% identity. When comparing the sequences derived from six plants of both the R and S populations, it was discovered that the R population plants contained a TGG to TGC mutation, causing a tryptophan to cysteine mutation at codon position 1999 on locus *Acc1;1*. This finding is in agreement with our previous report that the mutation Trp-1999-Cys was responsible for the resistance to ACCase-inhibiting herbicides [29].

## Discussion

In the present study, Japanese foxtail was determined to be a tetraploid weed by two commonly used methods, flow cytometer and chromosome counting. Flow cytometry is a convenient, fast and reliable method that has been used to determine the ploidy level of many species [32]. Since the reliability of flow cytometry compares with that of chromosome counting, this method has become widespread in biomedical research [32]. Here, flow cytometry offered a rapid method to determine the ploidy level of Japanese foxtail.

No difference of ploidy levels was observed between R and S populations, suggesting that resistance was irrelevant to ploidy level in this study. Puri et al. [35] found that there were ploidy variations among different biotypes of *Hydrilla verticillata* (Linn. f.) Royle, a serious problematic aquatic weed in the United States, with varying levels of resistance to fluridone. The differential resistance levels in different biotypes may also be due to ploidy variations [35]. Polyploidy is an important feature of evolution for many plant species [6], [36]. Polyploidy may confer new attributes, allowing plants to enter new environments or adapt to changing environments [6]. This may be significant to the weed's ability to evolve resistance under herbicide stress. Whether ploidy level can affect resistance in agricultural weeds is still unknown.

Multiple copies of the plastidic ACCase gene would be predicted based on the tetraploid nature of Japanese foxtail, and two copies of this gene were recently isolated by our group [29]. However, in the present study, the hybridization pattern after genomic DNA was digested with *Eco*RI, *Hae*III or both enzymes displayed two, three and three fragments, respectively. These results indicate the presence of at least two ACCase genes in Japanese foxtail. Similarly, when confirming the presence of multiple ALS genes in *Schoenoplectus mucronatus* (L.) Palla (three ALS genes have been isolated) by Southern blot, four fragments were observed [37]. The presence of at least three ALS genes in the *S. mucronatus* genome was concluded by Scarabel et al. [37]. In hexaploid *Avena sterilis* L. and *Avena fatua* Linn., three plastidic ACCase gene loci were isolated [8], [38]. In the tetraploid *Echinochloa phyllopogon* (Stapf.)Koss., four plastidic ACCase gene loci were isolated [39]. In addition, multiple copies of target genes have been observed in polyploid weed species when isolating the genes of ALS, which is another important herbicide-targeted enzyme [37], [39], [40], [41], [42]. Two partial cDNA sequences were isolated in this study. By comparing the sequences isolated from genomic DNA previously [29], we found that all of the homologous ACCase genes isolated were transcribed. Similarly, the three homologous ACCase genes in *A. fatua* were documented to be transcribed [8]. The mutation(s) in either of the ACCase gene loci may confer resistance since each gene was able to maintain its own mutation in Japanese foxtail and *A. fatua*. However, Iwakami et al. [39] reported that one of the four ACCase gene loci was not transcribed in *E. phyllopogon*. This may due to the pseudogenization of duplicated genes or gene silencing [43].

Although the ploidy levels of many plants have been documented, limited knowledge is available regarding that of agricultural weeds. For instance, the ploidy levels of the problematic weed species *Descurainia sophia* (Linn.) Webb. ex Prantl, *Myosoton aquaticum* (Linn.) Moench, *Galium aparine* Linn.and *P. fugax* are still unknown. In recent research, *D. sophia*, *M. aquaticum* and *G. aparine* were documented to confer resistance to tribenuron [44], [45], [46], [47], [48], and a population of *P. fugax* evolved resistance to ACCase-inhibiting herbicides [49]. Because of the unknown ploidy levels of these weeds, multiple copies of target genes may be ignored when they are studied. Even when the ploidy level was known for some polyploid weed species, multiple copies of target genes have been ignored [50], [51], [52]. Derived cleaved amplified polymorphic sequence (dCAPS), a simple technique to detect herbicide resistance-conferring gene mutations, has been widely used [8], [29], [53], [54], [55], [56]. This technique was considered able to detect heterozygous and homozygous loci with herbicide-resistant mutations. However, the presence of more than one target gene confounded the ability of this technique to accurately identify “true” heterozygotes [8], [29], [41]. Recently, we found that plants of the R population of Japanese foxtail carrying ACCase-inhibiting herbicide resistance-conferring mutations were always determined to be heterozygous using dCAPS [29], which suggested that multiple plastidic ACCase genes existed. The heterozygotes detected were probably homologous heterozygotes, not “true” allelic heterozygosity. These findings were in agreement with those of Yu et al. and Warwick et al. [8], [41]. In addition, the dilution effect could make the resistance more complex in polyploidy weed species [8], [39]. For multiple copies of target genes, the protein products of each gene can be diluted by the others. Under the same herbicide selection pressure, polyploid weed species may evolve resistance more slowly than diploids because of the dilution effect [39]. Relatively low-level resistance was observed in the polyploid *A. fatua* in contrast to high-level resistance conferred by the same mutations in unrelated diploid weed species [8]. This may in part be due to polyploidy and the dilution of resistance genes by susceptible genes. Thus, before studying the mechanism of resistance, the ploidy level and the target gene's copy number should be determined.

## References

[1] Bennett MD, Bhandol P, Leitch IJ (2000) Nuclear DNA amounts in angiosperms and their modern uses-807 new estimates. Ann Bot-London 86:859–909.

[2] Bennett MD, Leitch IJ (1995) Nuclear-DNA amounts in angiosperms. Ann Bot-London 76:113–176.

[3] Hanson L, McMahon KA, Johnson M, Bennett MD (2001) First nuclear DNA C-values for 25 angiosperm families. Ann Bot-London 87:251–258. 10.1006/anbo.2000.1325 32050741

[4] Bennett MD, Leitch IJ, Hanson L (1998) DNA amounts in two samples of angiosperm weeds. Ann Bot-London 82A:121–134.

[5] Green AF, Ramsey TS, Ramsey J (2013) Polyploidy and invasion of English ivy (*Hedera* spp., Araliaceae) in North American forests. Biol Invasions 15:2219–2241.

[6] Comai L (2005) The advantages and disadvantages of being polyploid. Nat Rev Genet 6:836–846. 16304599 10.1038/nrg1711

[7] Heap IM (2012) International Survey of Herbicide Resistant Weeds. Available: http://www.weedscience.org. Accessed 2014 May 8.

[8] Yu Q, Ahmad-Hamdani M, Han H, Christoffers MJ, Powles SB (2013) Herbicide resistance-endowing ACCase gene mutations in hexaploid wild oat (*Avena fatua*): insights into resistance evolution in a hexaploid species. Heredity 110:220–231. 23047200 10.1038/hdy.2012.69 PMC3668648

[9] Zhang HL, Yang ZR, Shen Y, Tong L (2003) Crystal structure of the carboxyltransferase domain of acetyl-coenzyme a carboxylase. Science 299:2064–2067. 12663926 10.1126/science.1081366

[10] Harwood JL (1988) Fatty-acid metabolism. Annu Rev Plant Physiol 39:101–138.

[11] Konishi T, Shinohara K, Yamada K, Sasaki Y (1996) Acetyl-CoA carboxylase in higher plants: Most plants other than gramineae have both the prokaryotic and the eukaryotic forms of this enzyme. Plant Cell Physiol 37:117–122. 8665091 10.1093/oxfordjournals.pcp.a028920

[12] Sasaki Y, Konishi T, Nagano Y (1995) The compartmentation of acetyl-coenzyme-a carboxylase in plants. Plant Physiol 108:445–449. 12228484 10.1104/pp.108.2.445 PMC157362

[13] Nikolau BJ, Ohlrogge JB, Wurtele ES (2003) Plant biotin-containing carboxylases. Arch Biochem Biophys 414:211–222. 12781773 10.1016/s0003-9861(03)00156-5

[14] Délye C (2005) Weed resistance to acetyl coenzyme A carboxylase inhibitors: an update. Weed Sci 53:728–746.

[15] Powles SB, Yu Q (2010) Evolution in action: Plants resistant to herbicides. Annu Rev Plant Biol 61:317–347. 20192743 10.1146/annurev-arplant-042809-112119

[16] Beckie HJ, Tardif FJ (2012) Herbicide cross resistance in weeds. Crop Prot 35:15–28.

[17] Délye C, Jasieniuk M, Le Corre V (2013) Deciphering the evolution of herbicide resistance in weeds. Trends Genet 29:649–658. 23830583 10.1016/j.tig.2013.06.001

[18] Kaundun SS, Bailly GC, Dale RP, Hutchings SJ, McIndoe E (2013) A novel W1999S mutation and non-target site resistance impact on acetyl-CoA carboxylase inhibiting herbicides to varying degrees in a UK *Lolium multiflorum* population. PLOS ONE 8(2):e58012 doi:10.1371/journal.pone.0058012 23469130 10.1371/journal.pone.0058012 PMC3585232

[19] Jang SR, Marjanovic J, Gornicki P (2013) Resistance to herbicides caused by single amino acid mutations in acetyl-CoA carboxylase in resistant populations of grassy weeds. New Phytol 197:1110–1116. 23301879 10.1111/nph.12117

[20] Liu WJ, Harrison DK, Chalupska D, Gornicki P, O'Donnell CC, et al (2007) Single-site mutations in the carboxyltransferase domain of plastid acetyl-CoA carboxylase confer resistance to grass-specific herbicides. Proc Natl Acad Sci USA 104:3627–3632. 17360693 10.1073/pnas.0611572104 PMC1802000

[21] Yu Q, Collavo A, Zheng MQ, Owen M, Sattin M, et al (2007) Diversity of acetyl-coenzyme a carboxylase mutations in resistant *Lolium* populations: Evaluation using clethodim. Plant Physiol 145:547–558. 17720757 10.1104/pp.107.105262 PMC2048730

[22] Délye C, Zhang XQ, Michel S, Matejicek A, Powles SB (2005) Molecular bases for sensitivity to acetyl-coenzyme a carboxylase inhibitors in black-grass. Plant Physiol 137:794–806. 15579665 10.1104/pp.104.046144 PMC1065379

[23] Clayton WD, Vorontsova MS, Harman KT, Williamson H GrassBase - The Online World Grass Flora. Available:http://www.kew.org/data/grasses-db.html. Accessed 2014 May 8.

[24] Xu H, Zhu X, Wang H, Li J, Dong L (2013) Mechanism of resistance to fenoxaprop in Japanese foxtail (*Alopecurus japonicus*) from China. Pestic Biochem Phys 107:25–31. 10.1016/j.pestbp.2013.04.008 25149231

[25] Bi YL, Liu WT, Li LX, Yuan GH, Jin T, et al (2013) Molecular basis of resistance to mesosulfuron-methyl in Japanese foxtail, *Alopecurus japonicus* . J Pestic Sci 38:74–77.

[26] Tang H, Li J, Dong L, Dong A, Lü B, et al (2012) Molecular bases for resistance to acetyl-coenzyme A carboxylase inhibitor in Japanese foxtail (*Alopecurus japonicus*). Pest Manag Sci 9:1241–1247. 10.1002/ps.3285 22461409

[27] Yang CH, Dong LY, Li J, Moss SR (2007) Identification of Japanese foxtail (*Alopecurus japonicus*) resistant to haloxyfop using three different assay techniques. Weed Sci 55:537–540.

[28] Mohamed IA, Li RZ, You ZG, Li ZH (2012) Japanese foxtail (*Alopecurus japonicus*) resistance to fenoxaprop and pinoxaden in China. Weed Sci 60:167–171.

[29] Xu H, Li J, Zhang D, Cheng Y, Jiang Y, et al. (2014) Mutations at codon position 1999 of acetyl-CoA carboxylase confer resistance to ACCase-inhibiting herbicides in Japanese foxtail (*Alopecurus japonicus*). Pest Manag Sci doi:10.1002/ps.3753. 10.1002/ps.3753 24497328

[30] Wang Y, Bigelow CA, Jiang YW (2009) Ploidy level and DNA content of perennial ryegrass germplasm as determined by flow cytometry. Hortscience 44:2049–2052.

[31] Amsellem L, Chevallier MH, Hossaert-McKey M (2001) Ploidy level of the invasive weed *Rubus alceifolius* (Rosaceae) in its native range and in areas of introduction. Plant Syst Evol 228:171–179.

[32] Dolězel J, Greilhuber J, Suda J (2007) Estimation of nuclear DNA content in plants using flow cytometry. Nat Protoc 2:2233–2244. 17853881 10.1038/nprot.2007.310

[33] Dolězel J, Binarova P, Lucretti S (1989) Analysis of nuclear DNA content in plant cells by flow cytometry. Biol Plantarum 31:113–120.

[34] Sieber VK, Murray BG (1979) The cytology of the genus *Alopecurus* (Gramineae). Bot J Linn Soc 79:343–355.

[35] Puri A, MacDonald GE, Haller WT (2007) Ploidy variations in floridone-susceptible and -resistant hydrilla (*Hydrilia verticillata*) biotypes. Weed Sci 55:578–583.

[36] Lafuma L, Balkwill K, Imbert E, Verlaque R, Maurice S (2003) Ploidy level and origin of the European invasive weed *Senecio inaequidens* (Asteraceae). Plant Syst Evol 243:59–72.

[37] Scarabel L, Locascio A, Furini A, Sattin M, Varotto S (2010) Characterisation of ALS genes in the polyploid species *Schoenoplectus mucronatus* and implications for resistance management. Pest Manag Sci 66:337–344. 19921713 10.1002/ps.1883

[38] Christoffers MJ, Berg ML, Messersmith CG (2002) An isoleucine to leucine mutation in acetyl-CoA carboxylase confers herbicide resistance in wild oat. Genome 45:1049–1056. 12502249 10.1139/g02-080

[39] Iwakami S, Uchino A, Watanabe H, Yamasue Y, Inamura T (2012) Isolation and expression of genes for acetolactate synthase and acetyl-CoA carboxylase in *Echinochloa phyllopogon*, a polyploid weed species. Pest Manag Sci 68:1098–1106. 22473865 10.1002/ps.3287

[40] Lamego FP, Charlson D, Delatorre CA, Burgos NR, Vidal RA (2009) Molecular basis of resistance to ALS-inhibitor herbicides in greater beggarticks. Weed Sci 57:474–481.

[41] Warwick SI, Sauder CA, Beckie HJ (2010) Acetolactate synthase (ALS) target-site mutations in ALS inhibitor-resistant Russian thistle (*Salsola tragus*). Weed Sci 58:244–251.

[42] Uchino A, Ogata S, Kohara H, Yoshida S, Yoshioka T, et al (2007) Molecular basis of diverse responses to acetolactate synthase-inhibiting herbicides in sulfonylurea-resistant biotypes of *Schoenoplectus juncoides* . Weed Biol Manag 7:89–96.

[43] Adams KL, Wendel JF (2005) Polyploidy and genome evolution in plants. Curr Opin Plant Biol 8:135–141. 15752992 10.1016/j.pbi.2005.01.001

[44] Cui HL, Zhang CX, Zhang HJ, Liu X, Liu Y, et al (2008) Confirmation of flixweed (*Descurainia sophia*) resistance to tribenuron in China. Weed Sci 56:775–779.

[45] Cui HL, Zhang CX, Wei SH, Zhang HJ, Li XJ, et al (2011) Acetolactate synthase gene Pro line (197) mutations confer tribenuron-methyl resistance in flixweed (*Descurainia sophia*) populations from China. Weed Sci 59:376–379.

[46] Sun JA, Wang JX, Zhang HJ, Liu JL, Bian SN (2011) Study on mutations in ALS for resistance to tribenuron-methyl in *Galium aparine* L. Agr Sci China 10:86–91.

[47] Liu W, Bi Y, Li L, Yuan G, Wang J (2013) Molecular basis of resistance to tribenuron in water starwort (*Myosoton aquaticum*) populations from China. Weed Sci 61:390–395.

[48] Liu WT, Bi YL, Li LX, Yuan GH, Du L, et al (2013) Target-site basis for resistance to acetolactate synthase inhibitor in Water chickweed (*Myosoton aquaticum* L.). Pestic Biochem Phys 107:50–54. 10.1016/j.pestbp.2013.05.003 25149235

[49] Tang W, Zhou FY, Chen J, Zhou XG (2014) Resistance to ACCase-inhibiting herbicides in an Asia minor bluegrass (*Polypogon fugax*) population in China. Pestic Biochem Phys 108:16–20. 10.1016/j.pestbp.2013.11.001 24485310

[50] Jin T, Liu JL, Huan ZB, Wu CX, Bi YL, et al (2011) Molecular basis for resistance to tribenuron in shepherd's purse (*Capsella bursa-pastoris* (L.) Medik.). Pestic Biochem Phys 100:160–164.

[51] Gherekhloo J, Osuna MD, De Prado R (2012) Biochemical and molecular basis of resistance to ACCase-inhibiting herbicides in Iranian *Phalaris minor* populations. Weed Res 52:367–372.

[52] Cui HL, Li XJ, Wang GQ, Wang JP, Wei SH, et al (2012) Acetolactate synthase proline (197) mutations confer tribenuron-methyl resistance in *Capsella bursa-pastoris* populations from China. Pestic Biochem Phys 102:229–232.

[53] Kaundun SS, Hutchings SJ, Dale RP, McIndoe E (2012) Broad resistance to ACCase inhibiting herbicides in a ryegrass population is due only to a cysteine to arginine mutation in the target enzyme. PLOS ONE 7(6):e39759 doi:10.1371/journal.pone.0039759 22768118 10.1371/journal.pone.0039759 PMC3387263

[54] Délye C, Pernin F, Michel S (2011) 'Universal' PCR assays detecting mutations in acetyl-coenzyme A carboxylase or acetolactate synthase that endow herbicide resistance in grass weeds. Weed Res 51:353–362.

[55] Kaundun SS (2010) An aspartate to glycine change in the carboxyl transferase domain of acetyl CoA carboxylase and non-target-site mechanism(s) confer resistance to ACCase inhibitor herbicides in a *Lolium multiflorum* population. Pest Manag Sci 66:1249–1256. 20648527 10.1002/ps.2003

[56] Kaundun SS, Windass JD (2006) Derived cleaved amplified polymorphic sequence, a simple method to detect a key point mutation conferring acetyl CoA carboxylase inhibitor herbicide resistance in grass weeds. Weed Res 46:34–39.

